# Injury of Macrophages Induced by *Clostridium perfringens* Type C Exotoxins

**DOI:** 10.3390/ijms25073718

**Published:** 2024-03-27

**Authors:** Siyu Zhang, Dong Wang, Yawen Ding, Fuyang Song, Yong Li, Jin Zeng, Yujiong Wang

**Affiliations:** Key Laboratory of Ministry of Education for Conservation and Utilization of Special Biological Re-Sources in the Western China, College of Life Science, Ningxia University, Yinchuan 750021, China; zhangsiyu1997@163.com (S.Z.); dongw1998@163.com (D.W.); 15226260103@163.com (Y.D.); songfy26@163.com (F.S.); liyong7732@nxu.edu.cn (Y.L.)

**Keywords:** *Clostridium perfringens*, exotoxins, oxidative stress, ferroptosis, macrophages

## Abstract

*Clostridium perfringens* is a kind of anaerobic Gram-positive bacterium that widely exists in the intestinal tissue of humans and animals. And the main virulence factor in *Clostridium perfringens* is its exotoxins. *Clostridium perfringens* type C is the main strain of livestock disease, its exotoxins can induce necrotizing enteritis and enterotoxemia, which lead to the reduction in feed conversion, and a serious impact on breeding production performance. Our study found that treatment with exotoxins reduced cell viability and triggered intracellular reactive oxygen species (ROS) in human mononuclear leukemia cells (THP-1) cells. Through transcriptome sequencing analysis, we found that the levels of related proteins such as heme oxygenase 1 (HO-1) and ferroptosis signaling pathway increased significantly after treatment with exotoxins. To investigate whether ferroptosis occurred after exotoxin treatment in macrophages, we confirmed that the protein expression levels of antioxidant factors glutathione peroxidase 4/ferroptosis-suppressor-protein 1/the cystine/glutamate antiporter solute carrier family 7 member 11 (GPX4/FSP1/xCT), ferroptosis-related protein nuclear receptor coactivator 4/transferrin/transferrin receptor (NCOA4/TF/TFR)/ferritin and the level of lipid peroxidation were significantly changed. Based on the above results, our study suggested that *Clostridium perfringens* type C exotoxins can induce macrophage injury through oxidative stress and ferroptosis.

## 1. Introduction

In early investigations, *Clostridium perfringens* was divided into five types, A, B, C, D and E, according to the main lethal toxins. It was expanded to seven types (A–G) in 2017 [1]. Among them, type C has strong virulence and is the main pathogenic strain of disease in cultured animals. This bacterium causes animal intestine necrosis, which is difficult to prevent and treat, and has a wide epidemic range, thus attracting a lot of attention [2]. It can also cause foodborne illness in humans; the reason is usually that the patients eat raw meat and its products contaminated by the bacterium. These contaminated foods cause a large number of reproductions of the bacterium and secrete exotoxins due to improper processing and storage [3]. And its harmfulness in livestock and poultry breeding production is of greater concern. Especially in recent years, with the implementation of the “Antibiotic Prohibition Order” and the discontinuation of antibiotic growth promoters in animal feed, the incidence rate of necrotizing enteritis caused by this bacterium has increased significantly [4]. The exotoxins produced by the bacterium can affect almost all farmed animals, such as cattle, sheep, pigs and birds. Animals often get sick quickly in a short time, leading to shock and even death. The disease has a particularly serious impact on young animals. Even young animals that survive the treatment of a large number of antibiotics often have subclinical phenomena of growth retardation. Subclinical infection can weaken the growth rate of livestock and poultry, reduce feed conversion rate and weight gain, seriously affect their breeding and production performance, and lead to significant economic losses [5]. It is reported that *Clostridium perfringens* type C causes about USD 6 billion of production losses and control costs to the global poultry industry every year [6,7].

*Clostridium perfringens* uses a large number of toxins secreted by it to cause intestinal infection, tissue toxicity or other neurological reactions in humans and animals. It is generally believed that, under some conditions, humans or animals ingest *Clostridium perfringens*, or the bacteria adhering to the inner wall of the digestive tract overmultiply, rapidly producing a variety of toxins and invasive enzymes. Exotoxins act on receptors on a variety of target cells in the intestinal tract, activating intracellular signal pathways and then producing various effects. The susceptible cells of exotoxins include macrophages, endothelial cells, etc. When exotoxins act on the target cell, they will combine with the cell plasma membrane, destroy the ion balance of the internal and external environment of the cell, and cause the cell to swell and crack. It should be noted that depletion of intracellular adenosine triphosphate (ATP), membrane lipid damage and/or loss of ATP-dependent ion pump function will cause loss of cell membrane potential, leading to swelling of organelles, mitochondrial damage and plasma membrane rupture. Mitochondria are the main site of reactive oxygen species (ROS) production in most cells and, when mitochondria are damaged, toxic levels of ROS will accumulate. At higher ROS levels, longer mitochondrial permeability transition pore (mPTP) openings may release an ROS burst, leading to destruction of mitochondria [8]. Moreover, ferroptosis is a type of programmed cell death that is dependent on iron and the accumulation of lipid peroxides, which cause damage to the lipid bilayer of the plasma membrane through accelerated oxidation of membrane lipids [9]. The excessive production of ROS and the activation of lipid peroxidation are linked to ferroptosis [10]. Mitochondrial damage has been found to be involved in lipid peroxidation and imbalance of iron homeostasis in some diseases [11]. Based on this, whether exotoxins cause oxidative stress by accumulating ROS, leading to mitochondrial damage and ferroptosis, resulting in cell damage, needs further research.

Therefore, this study directly used culture supernatant of type C in brain heart infusion to infect cells. The changes were analyzed by detecting cell viability, transcriptome sequencing analysis, the level of ROS, lipid peroxidation, glutathione (GSH) and malonaldehyde (MDA), the expression of antioxidant-related factors and ferroptosis-related protein. The differentially expressed genes (DEGs) were screened and the key signal pathways were screened through gene ontology (GO) function annotation and Kyoto encyclopedia of genes and genomes (KEGG) pathway enrichment to provide a theoretical basis for the study of the pathogenic mechanism of exotoxins secreted by *Clostridium perfringens* type C.

## 2. Results

### 2.1. CP-BHI Induced the Death of Macrophages

**Figure 1:**
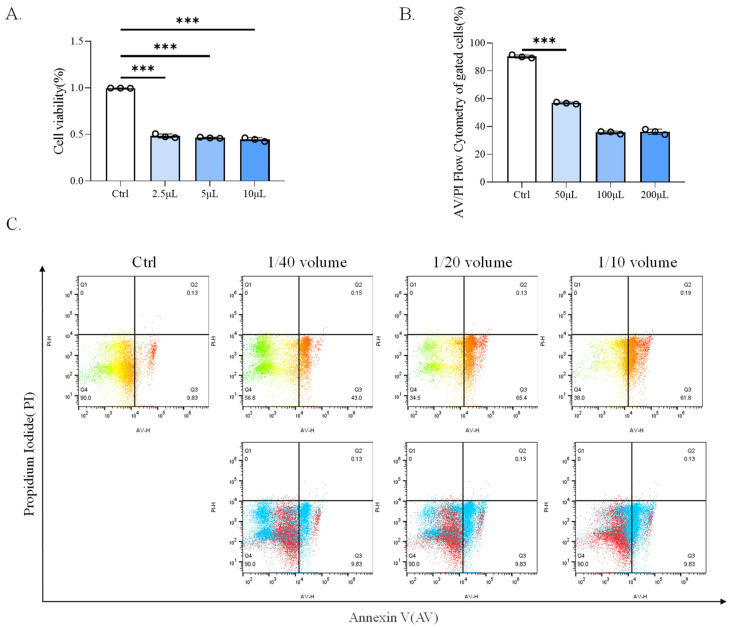
CP-BHI induced the death of macrophages. (**A**) The cell viability of THP-1 cells treated with different concentrations of CP-BHI for 8 h was analyzed by the CCK-8 test. The control cells were calibrated as 100%. (**B**,**C**) FACS detection of Annexin-V/PI analysis of THP-1 cells treated with different concentrations of CP-BHI for 8 h. *p* < 0.001 *** indicates statistically significant differences.

To investigate the 50% inhibitory concentration (IC50) in human mononuclear leukemia cells (THP-1) viability, different concentrations of CP-BHI were added in cells. As shown in Figure 1A, cells exposed to CP-BHI significantly decreased at a concentration of 1/40 volume. Therefore, this concentration was used in further study. In addition, we obtained consistent results through Annexin-V/PI assay (Figure 1B,C). These data showed that CP-BHI could induce the death of the cells.

### 2.2. CP-BHI Induced ROS Production in Macrophages

**Figure 2:**
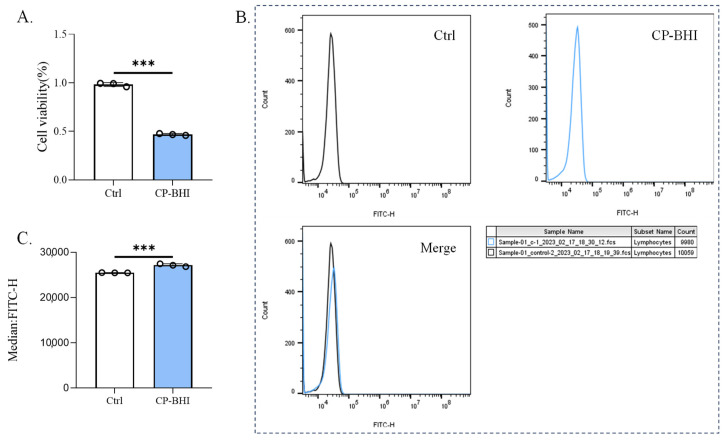
CP-BHI induced ROS production in macrophages. (**A**) The cell viability of THP-1 cells treated with 1/40 volume of CP-BHI for 8 h was analyzed by the CCK-8 test. The control cells were calibrated as 100%. (**B**,**C**) FACS detection of ROS analysis of THP-1 cells treated with 1/40 volume of CP-BHI for 8 h; the three “diagram of curves” belong to Figure 2B. *p* < 0.001 *** indicates statistically significant differences.

After THP-1 cells were incubated for 8 h with 1/40 volume of CP-BHI, the cell viability was analyzed. As shown in Figure 2A, cells were lower than the control group. This result suggested that the culture supernatant with cytotoxicity was successfully prepared. To investigate whether CP-BHI induced the production of ROS, DCFH-DA was added to cells. After THP-1 cells were incubated for 8 h with 1/40 volume of CP-BHI, the ROS were evaluated in comparison with the control group (Figure 2B,C). These data showed that CP-BHI induced the accumulation of ROS in macrophages.

### 2.3. CP-BHI Decreased the Expression of Antioxidant-Related Factors in Macrophages

**Figure 3:**
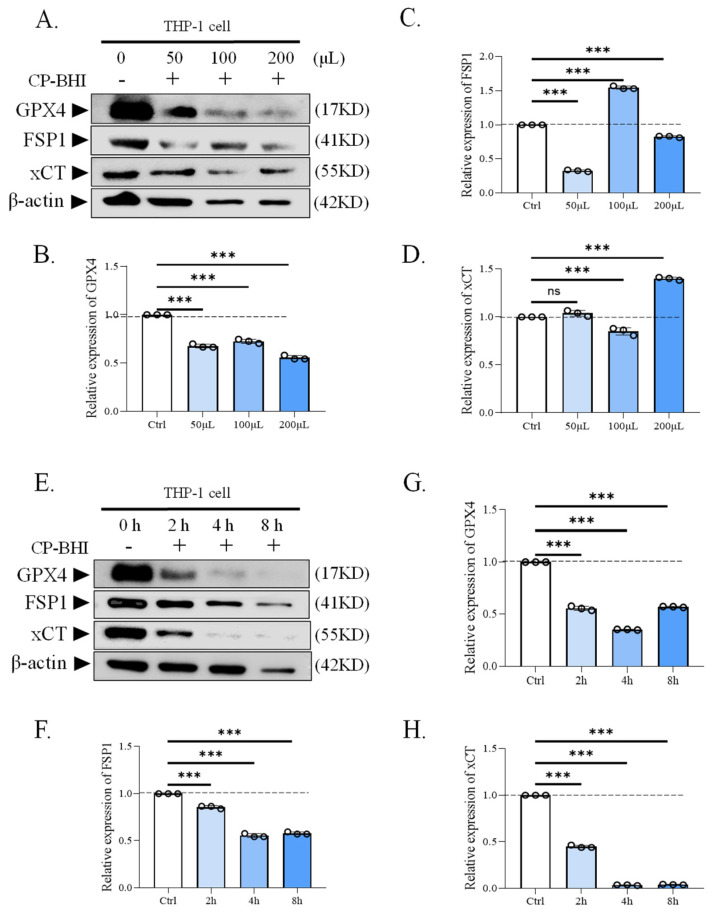

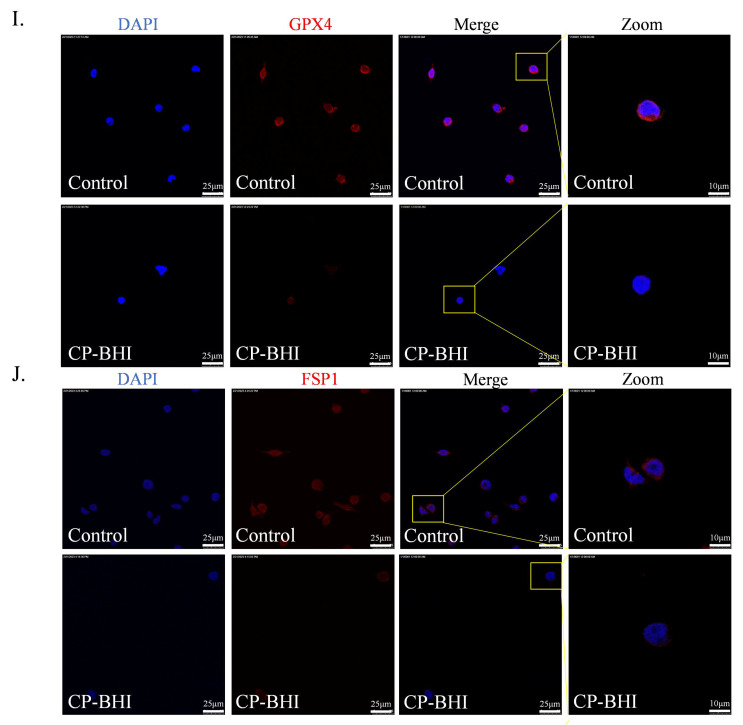
CP-BHI decreased the expression of antioxidant-related factors in macrophages. (**A**) The expression of antioxidant-related factors in THP-1 cells treated with different concentrations of CP-BHI was analyzed by Western blotting. (**B**,**G**) The semi-quantification of GPX4. (**C**,**F**) The semi-quantification of FSP1. (**D**,**H**) The semi-quantification of xCT. (**E**) The representative blots of cells treated with CP-BHI for different times. (**I**) Representative immunofluorescence images of GPX4. (**J**) Representative immunofluorescence images of FSP1. *p* < 0.001 *** indicates statistically significant differences, and ns indicates no significant difference.

To investigate whether CP-BHI led to the collapse of the antioxidant system through the accumulation of ROS, the expression of antioxidant-related factors was analyzed, such as antioxidant factors glutathione peroxidase 4/ferroptosis-suppressor-protein 1/the cystine/glutamate antiporter solute carrier family 7 member 11 (GPX4/FSP1/xCT). The results of western blotting (Figure 3A–H) and immunofluorescent staining assay (Figure 3I,J) demonstrated a decrease in antioxidant-related factors in macrophages treated with CP-BHI. These data indicated that CP-BHI induced the collapse of the pro-/antioxidant balance through the excessive accumulation of ROS.

### 2.4. Transcriptome Analysis of THP-1 Cells Treated with CP-BHI Revealed Differential Expression of Ferroptosis-Related Genes

**Figure 4:**
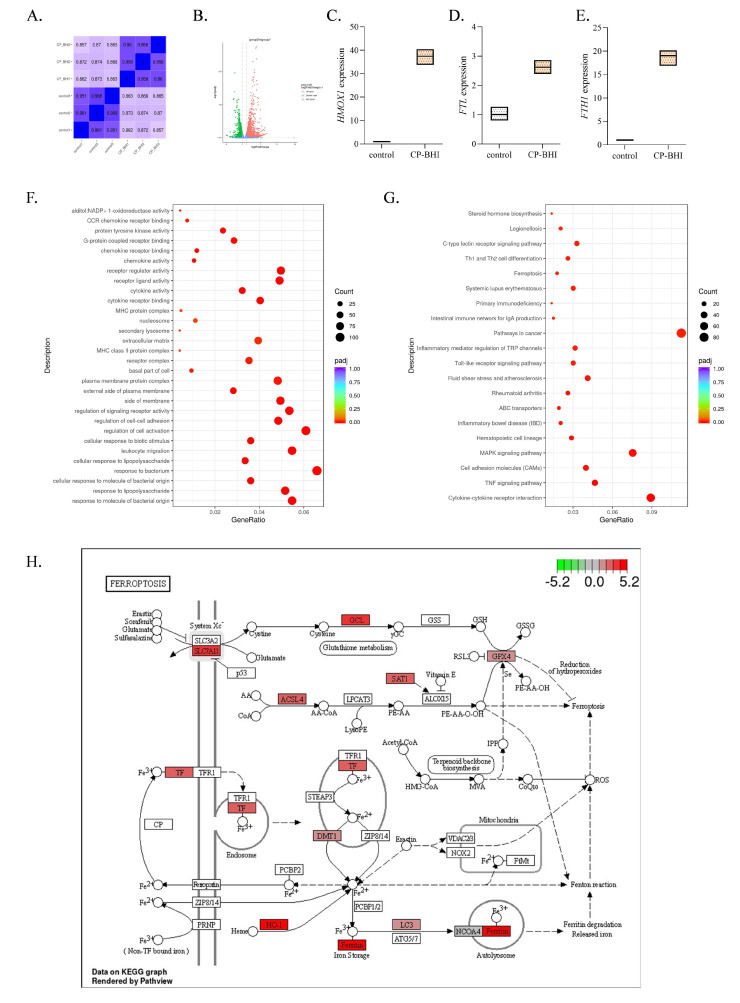
CP-BHI could induce ferroptosis in THP-1 cells. (**A**) The heat map of correlation between samples. (**B**) Volcanic map. (**C**) The expression of *HO-1*. (**D**) The expression of *FTL*. (**E**) The expression of *FTH1*. (**F**) GO database annotation of DEGs. (**G**) KEGG enrichment analysis of DEGs. (**H**) The plot of KEGG.

To investigate whether ferroptosis is involved in THP-1 cells treated with CP-BHI, cells were collected for transcriptome analysis. After filtering and sorting out the original data, effective data were obtained (Figure 4A, Supplementary Table S1), which could be used for subsequent analysis. As shown in the volcanic map in Figure 4B, there were 2547 genes upregulated and 1324 genes downregulated, compared with the control group. Among them, heme oxygenase 1 (*HO-1*) (Figure 4C), *ferritin heavy chain 1* (*FTH1*) (Figure 4D) and *ferritin light chain (FTL)* (Figure 4E) were upregulated. The DEGs were enriched by GO function, as shown in Figure 4F; NADP+1-oxidoreductase activity was significantly different. The enrichment results of the KEGG pathway showed that (Figure 4G) ferroptosis signaling pathway was significantly different. Moreover, transferrin (TF), HO-1 and ferritin showed a significant increase in the plot of KEGG (Figure 4H). These data indicated that CP-BHI could induce ferroptosis in THP-1 cells.

### 2.5. CP-BHI Increased the Expression of Ferroptosis-Related Protein in Macrophages

**Figure 5:**
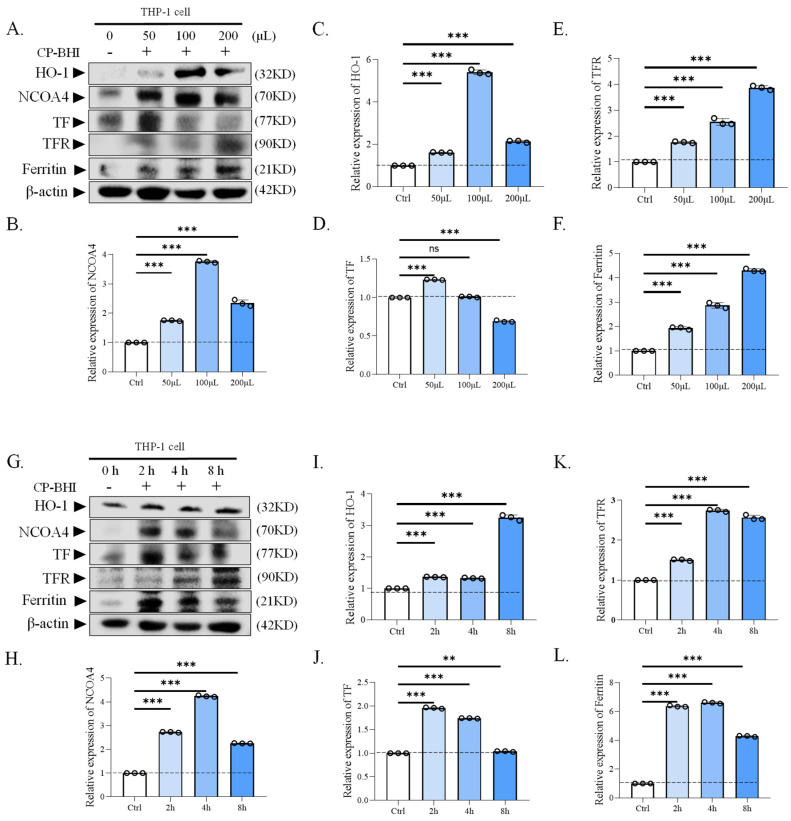
CP-BHI increased the expression of ferroptosis-related protein in macrophages. (**A**) The expression of ferroptosis-related protein in THP-1 cells treated with different concentrations of CP-BHI was analyzed by western blotting. (**B**,**H**) The semi-quantification of NCOA4. (**C**,**I**) The semi-quantification of HO-1. (**D**,**J**) The semi-quantification of TF. (**E**,**K**) The semi-quantification of TFR. (**F**,**L**) The semi-quantification of ferritin. (**G**) The representative blots of cells treated with CP-BHI for different times. *p* < 0.01 **, *p* < 0.001 *** indicates statistically significant differences, and ns indicates no significant difference.

To verify that CP-BHI induces ferroptosis in macrophages, the expression of ferroptosis-related proteins was analyzed, such as HO-1, ferroptosis-related protein nuclear receptor coactivator 4, transferrin, transferrin receptor (NCOA4/TF/TFR) and ferritin. The results of western blotting demonstrated an increase in ferroptosis-related protein in macrophages treated with CP-BHI (Figure 5A–L). These data indicated that CP-BHI could induce ferroptosis in macrophages through the NCOA4/HO-1/TF/TFR/ferritin axis.

### 2.6. CP-BHI Increased the Level of Lipid Peroxidation in Macrophages

**Figure 6:**
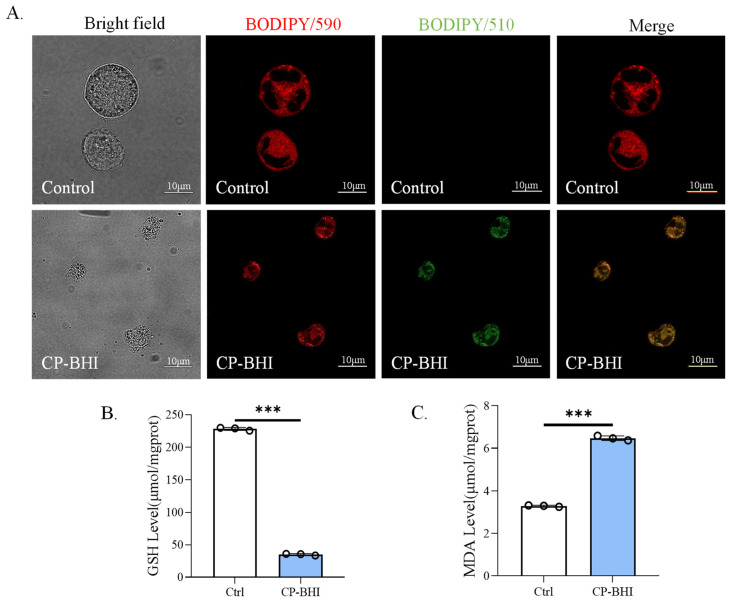
CP-BHI increased the level of lipid peroxidation in macrophages. (**A**) Representative images of BODIPY 581/591 C11-labeled lipoxidation of polyunsaturated fatty acids. (**B**) The level of GSH. (**C**) The level of MDA. *p* < 0.001 *** indicates statistically significant differences.

We verified the level of lipid peroxidation after treatment with CP-BHI. As shown in Figure 6A, CP-BHI-infected THP-1 cells showed a strong positive lipid peroxidation (green/510 nm) compared to the uninfected cells. In addition, the level of GSH in the CP-BHI-infected group was lower than the control group (Figure 6B), while the level of MDA in the CP-BHI-infected group was greater than the control group (Figure 6C). These data indicated that an increase in lipid peroxidation induces ferroptosis in macrophages when treated with CP-BHI.

## 3. Discussion

*Clostridium perfringens* is the major causative agent of enteritis necroticans and enterotoxemia in animals and it can cause food poisoning in humans [12,13]. In recent years, the use of antibiotics has been banned, making it urgent to investigate molecular therapy options for diseases caused by this bacterium. The main virulence factors of this bacterium are its exotoxins. Among these exotoxins, α and β are the most important pathogenic factors of *Clostridium perfringens* type C. α-toxin (CPA) has phospholipase and sphingomyelinase activities, and lytic concentrations of CPA can result in extensive degradation of plasma membrane, lactate dehydrogenase (LDH) release and generation of ROS [14,15]. β-toxin, also known as β1-toxin, rapidly induces cellular events consistent with necrosis, including LDH release [16,17]. And results indicated that rCPB2 toxin induces apoptosis and inflammation in IPEC-J2 cells [18]. The above research suggested that exotoxins of *Clostridium perfringens* type C induce cell damage. In addition, since this bacterium produces many exotoxins and it is difficult to isolate a single toxin for research, we directly used the culture supernatant to attack cells and study the pathogenic mechanism of type C strains.

*Clostridium perfringens* is commonly found in intestinal tracts, and its exotoxins are mainly absorbed via the intestinal mucosa, causing disease development [19]. In some studies, the MTS assay was performed with five immune cell lines to investigate the cytotoxicity of β1-toxin. The results indicated that U937 and THP-1 cells are highly sensitive [20]. In our research, THP-1 cells were chosen to study the pathogenic mechanism of type C strains. And our results showed that exotoxins could induce the death of the THP-1 cells. In addition, there are studies that verified that, following exposure to *Clostridium perfringens* phospholipase C (CpPLC), a significant generation of mitochondrial ROS was observed, and these disturbances further led to alterations in the mitochondrial genome and functioning [21]. Moreover, the levels of ROS showed a significant increase in CPB2 toxin-induced IPEC-J2 cells [22]. And epsilon toxin inhibits mitochondrial function and induces ROS and lipid membrane damage [23]. Based on this, to investigate whether *Clostridium perfringens* type C exotoxins induce the production of ROS and oxidative stress, we performed DCFDA staining to verify it. ROS is crucial in determining cell fate. Under normal conditions, antioxidants can counteract free radicals and neutralize oxidants, protecting cells from ROS-induced lipid peroxidation, such as glutathione peroxidase (GPX). If ROS is excessively produced and accumulated, oxidants and antioxidants will be out of balance, leading to oxidative stress (OS), and cells also will be irreversibly damaged. Our results showed that antioxidant-related factors were decreased in macrophages treated with exotoxins, including GPX4, FSP1 and xCT. The results indicated that exotoxins induced the collapse of the pro-/antioxidant balance through the excessive accumulation of ROS. It is worth noting that ROS is converted into H_2_O_2_ through superoxide dismutase (SOD) and produces highly toxic hydroxyl in the presence of reduced iron (Fe^2+^) through Fenton reaction. And Fe^2+^ triggers ferroptosis via Fenton reactions and ROS accumulation [24]. In addition, GPX4 prevents ferroptosis by converting lipid hydroperoxides into nontoxic lipid alcohols [25,26]. And FSP1 is a potent ferroptosis suppressor [27]. All the changes indicated that exotoxins could induce ferroptosis in THP-1 cells. 

Ferroptosis, driven by iron-dependent phospholipid peroxidation, is regulated by multiple cellular metabolic events, including redox homeostasis, iron handling, mitochondrial activity, and metabolism of amino acids, lipids and sugars, in addition to numerous signaling pathways relevant to disease [28]. Increasing numbers of studies have shown that high expression of HO-1 is an important positive regulator of ferroptosis, which makes it an important candidate to mediate detrimental effects such as ferroptosis induction [29]. Apparently, ferroptosis can be induced by HO-1 through regulation of the amount of cellular iron and ROS. Moreover, recent studies demonstrate that ferritin is a key ferroptotic regulator, and its level affects the susceptibility to ferroptosis in vitro and in vivo [30]. In ferritinophagy, NCOA4 directly recognizes and binds FTH1, then delivers iron-bound ferritin to autophagosomes for lysosomal degradation and iron release. And it has been reported that ferritinophagy participates in ferroptosis. The knockdown of NCOA4 can inhibit ferritinophagy and block lipid peroxidation and ferroptosis by reducing the amount of bioavailable intracellular labile iron pools [31,32]. To investigate the mechanism of damage in THP-1 cells by exotoxins-induced ferroptosis, we collected cells for transcriptome analysis. Consistent with the above research, our results showed that HO-1, FTH1 and FTL were upregulated and NADP+1-oxidoreductase activity and ferroptosis signaling pathway were significantly different. Moreover, TF, HO-1 and ferritin showed a significant increase in the plot of KEGG. Iron is mainly taken up into cells via TFR, which transports TF-bound iron through receptor-mediated endocytosis. Interestingly, TFR was identified and validated as a ferroptosis marker among other proposed biomarkers [33]. Consistent with this, our results showed that the expression of ferroptosis-related protein was increased in macrophages treated with exotoxins, including HO-1, NCOA4, TF, TFR and ferritin. These results suggested autophagy-dependent ferroptosis occurred in THP-1 cells exposed to exotoxins. In addition, lipid peroxidation was also validated as a ferroptosis marker. Our results showed that the level of GSH in the exotoxins-infected group was decreased, while the level of lipid peroxidation and MDA in the exotoxins-infected group were increased. These data indicated that an increase in lipid peroxidation induces ferroptosis in macrophages when treated with exotoxins.

## 4. Materials and Methods

### 4.1. Bacterial Strains, Cell Lines, and Reagents

The Chinese standard strain of *Clostridium perfringens*, C59-2 (type C), was kept by our laboratory. THP-1 were purchased from the National Collection of Authenticated Cell Cultures. The brain heart infusion (BHI) medium was purchased from OXOID, Cheshire, UK.

### 4.2. Cell Culture and Treatment

The THP-1 cells were cultured in RPMI 1640 with 10% fetal bovine serum (FBS) in a humidified 5% CO_2_ atmosphere at 37 °C. Then, the cells in good condition were transferred to a six-well plate at a density of 1 × 10^6^ cells (volume: 2 mL)/well or a 96-well plate at a density of 8 × 10^3^ cells (volume: 100 μL)/well and cultured overnight. C59-2 strain was cultured in BHI medium until the fourth generation. Then, the bacterial solution was centrifuged and passed through a 0.22 μm filter to obtain supernatant (CP-BHI). CP-BHI was added to the cells for different times and concentrations before analysis. 

### 4.3. Cell Viability

Cells were seeded in a six-well plate at 1 × 10^6^ cells/well (Annexin-V/PI assay) or a 96-well plate at a density of 8 × 10^3^ cells/well (CCK-8 assay) and treated with CP-BHI for different concentrations (1/40 volume, 1/20 volume and 1/10 volume). Then, cell viability was measured using the Annexin-V/PI kit (Solarbio, Beijing, China) or CCK-8 kit (biosharp, Hefei, China) according to the manufacturer’s recommendations. CCK-8 solution was added to each well. After 1.5 h, the absorbance value was measured at 450 nm using a luciferase plate analyzer and the data were recorded.

### 4.4. Measurement of Reactive Oxygen Species (ROS)

Cells were seeded in a six-well plate at a density of 1 × 10^6^ cells/well and treated with CP-BHI. Then, ROS was measured using the assay kit according to the manufacturer’s recommendations (Biorigin, Beijing, China). DCFH-DA (10 mM) was added to each well, with incubation at 37 °C for 30 min. The cells were then washed with PBS and the fluorescence intensity was detected by using flow cytometry.

### 4.5. Transcriptome Sequencing Analysis

Cells were seeded in a 10 cm dish at a density of 1 × 10^7^ and treated with CP-BHI. Then, the cell samples were sent to Nuohe Zhiyuan Technology Co., Ltd. (Beijing, China), to sequence using Nova seq on the Illumina 2000 sequencing platform. After raw data filtering, sequencing error rate check, and GC-content distribution check, clean reads for subsequent analysis were obtained [34]. And, after filtering out unqualified data, Cluster Profiler version 4.00 software was used to perform GO analysis [35,36] and KEGG pathway analysis [37] on the differential gene set to screen for related signaling molecular pathways that play a crucial role in biological processes.

### 4.6. Immunoblotting

Cells were seeded in a six-well plate at a density of 1 × 10^6^ cells/well and treated with CP-BHI. Then, cells were dissociated by the total protein extraction kit (KeyGEN, Nanjing, China). The proteins in the samples were run on 10% SDS polyacrylamide gel and then transferred to polyvinylidene fluoride membrane (Millipore, St. Louis, MO, USA). The proteins were incubated with primary antibody (Abmart, Shanghai, China) overnight at 4 °C and secondary anti-rabbit HRP-conjugated antibodies (Abmart, China) for 1 h at RT. Then, the membranes were developed in chemiluminescence. The primary antibodies used were listed as follows: GPX4, FSP1, xCT, NCOA4, HO-1, TF, TFR, ferritin and β-actin.

### 4.7. Immunofluorescent Staining

Cells were seeded in a 12-well plate at a density of 3 × 10^5^ cells/well and treated with CP-BHI. Then, cells were washed in phosphate buffer solution (PBS) for 3 × 5 min, fixed in 4% paraformaldehyde for 30 min at RT, penetrated with 0.1% Triton X-100 for 20 min at RT and blocked with 5% bovine serum albumin (BSA) for 1 h at RT, followed by incubating in primary antibody (Abmart, China) overnight at 4 °C and fluorescent secondary antibody (Abmart, China) without light for 1 h at RT. Cell nuclei were counterstained with DAPI. The fluorescence intensity was detected by using laser scanning confocal microscope (SP5, Leica, Wetzlar, Germany).

### 4.8. Detection of Lipid Peroxidation Level

Cells were seeded in a six-well plate at a density of 1 × 10^6^ cells/well and treated with CP-BHI. Then, cellular lipid peroxidation level was measured using the Image-iT^®^ Lipid Peroxidation Kit according to the manufacturer’s recommendations (ThermoFisher, Waltham, MA, USA). BODIPY^®^ 581/591 C11 reagent was added to each well, with incubation at 37 °C for 30 min. The cells were then washed with PBS and the fluorescence intensity was detected by using a laser scanning confocal microscope.

### 4.9. Detection of GSH and MDA Level

Cells were seeded in a six-well plate at a density of 1 × 10^6^ cells/well and treated with CP-BHI. Then, cellular GSH or MDA level was measured using the assay kit according to the manufacturer’s recommendations (Beyotime, Haimen, China). The absorbance value was measured at 532 nm using a luciferase plate analyzer and the data were recorded. 

### 4.10. Statistical Analysis

All data were obtained after at least three independent experiments. One-way ANOVA in GraphPad Prism 7.0 software was used for statistical analysis in three or more groups, while t-test was used in two groups. A difference was considered to be statistically significant at a *p* value of <0.05. Data are presented as the mean ± standard deviation (SD).

## 5. Conclusions

**Figure 7:**
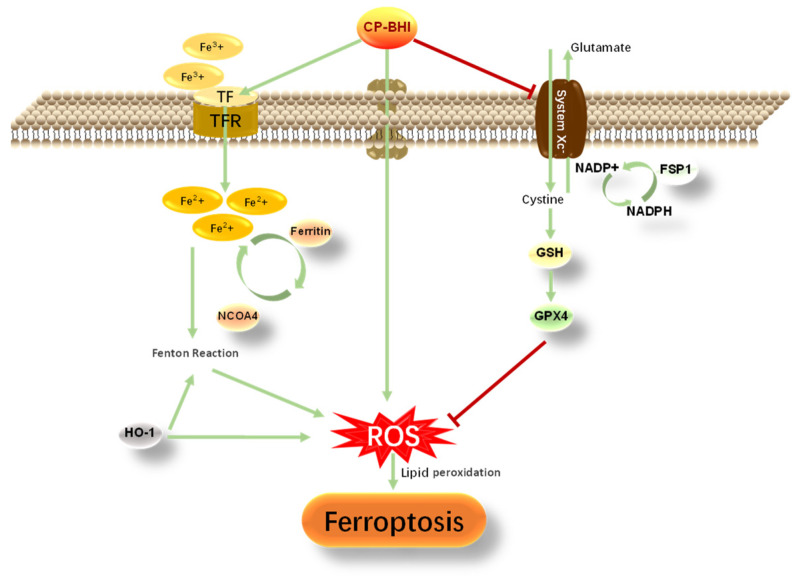
The mechanism by which *Clostridium perfringens* type C exotoxins promotes oxidative stress and ferroptosis in macrophages.

In conclusion, *Clostridium perfringens* type C exotoxins induce the accumulation of ROS and decrease in antioxidant-related factors, induce the collapse of the pro-/antioxidant balance and cause oxidative stress. In addition, *Clostridium perfringens* type C exotoxins induce iron overload, leading to an increase in Fenton reaction, and induce the production of ROS. Excessive ROS increases the level of lipid peroxidation, resulting in ferroptosis and injury in macrophages (Figure 7).

## Data Availability

All relevant data are either presented within the manuscript or made available online as Supplementary Materials. The information is accessible upon reasonable request. The study’s raw data and associated analyses may be obtained from the corresponding author upon reasonable request.

## Supplementary Materials

The following are available online at https://www.mdpi.com/article/10.3390/ijms25073718/s1.
